# Modeling *Mycobacterium tuberculosis* early granuloma formation in experimental human lung tissue

**DOI:** 10.1242/dmm.013854

**Published:** 2013-11-07

**Authors:** Venkata Ramanarao Parasa, Muhammad Jubayer Rahman, Anh Thu Ngyuen Hoang, Mattias Svensson, Susanna Brighenti, Maria Lerm

**Affiliations:** 1 Center for Infectious Medicine, Department of Medicine, Karolinska Institute, Stockholm 14186, Sweden.; 2 Division of Microbiology and Molecular Medicine, Department of Clinical and Experimental Medicine, Linköping University, Linköping 58185, Sweden.

**Keywords:** Tuberculosis, *M. tuberculosis*, Granuloma, *In vitro* model, Lung tissue, ESX-1

## Abstract

The widely used animal models for tuberculosis (TB) display fundamental differences from human TB. Therefore, a validated model that recapitulates human lung TB is attractive for TB research. Here, we describe a unique method for establishment of TB infection in an experimental human lung tissue model. The model is based on cell lines derived from human lungs and primary macrophages from peripheral blood, and displays characteristics of human lung tissue, including evenly integrated macrophages throughout the epithelium, production of extracellular matrix, stratified epithelia and mucus secretion. Establishment of experimental infection in the model tissue with *Mycobacterium tuberculosis*, the bacterium that causes TB, resulted in clustering of macrophages at the site of infection, reminiscent of early TB granuloma formation. We quantitated the extent of granuloma formation induced by different strains of mycobacteria and validated our model against findings in other TB models. We found that early granuloma formation is dependent on ESAT-6, which is secreted via the type VII secretion machinery of virulent mycobacteria. Our model, which can facilitate the discovery of the interactions between mycobacteria and host cells in a physiological environment, is the first lung tissue model described for TB.

## INTRODUCTION

Tuberculosis (TB) is caused by *Mycobacterium tuberculosis* (Mtb), which is one of the most successful pathogens in the world. TB claims 1.2 million human lives annually (Lozano et al., 2012) and the HIV epidemic fuels its spread, because HIV infection dramatically increases the risk of developing active TB. Multidrug-resistant TB (MDR-TB) is on the rise – in some parts of the world, 30% of new cases are MDR-TB – leaving the patients with limited options for cure.

Mtb is a highly successful intracellular pathogen that has evolved several strategies to evade killing and persist inside human macrophages even in the presence of a host immune response (Abramovitch et al., 2011; Welin et al., 2011b; Velmurugan et al., 2007). Active replication of Mtb inside the macrophage results in killing of the host cell (Abdallah et al., 2011; Welin et al., 2011a). However, when exposed to stress, Mtb has the ability to switch phenotype to a non-replicating form suitable for persistence (Deb et al., 2009). A well-known virulence factor of Mtb is early secreted antigenic target of 6 kDa (ESAT-6), which is secreted via the mycobacterial type VII secretion system (ESX-1). Recent evidence suggests that ESX-1-mediated translocation of Mtb from the phagolysosome to the macrophage cytoplasm is a central virulence mechanism of pathogenic mycobacteria (Abdallah et al., 2011; Houben et al., 2012). Both the ESX-1 components and ESAT-6 are encoded in the region of difference 1 (RD1) and are present in virulent Mtb but absent or dysfunctional in avirulent strains (Frigui et al., 2008). Granulomas, which are hallmarks of human TB, are organized tissue structures composed of clusters of infected macrophages and multinucleated giant cells, surrounded by aggregates of newly recruited monocytes/macrophages, neutrophils and lymphocytes (Brighenti and Andersson, 2012; Flynn et al., 2011). The relationship between RD1, the ability of *Mycobacterium marinum* to induce granuloma formation and mycobacterial dissemination was demonstrated by Ramakrishnan and colleagues in an elegant study performed in the zebrafish embryo model (Davis and Ramakrishnan, 2009). However, the early events leading to granuloma formation in human TB are unclear and, therefore, well-characterized human models are required to study these mechanisms.

To date, there are no existing *in vitro* models for human TB, with the exception of infection of cells isolated from human blood (Kapoor et al., 2013; Puissegur et al., 2004). Most experimental TB research is being done in mice, although the mouse immune response to TB infection is fundamentally different from humans (Gupta and Katoch, 2005). Other commonly used animal models include guinea pig (Kashino et al., 2008), rabbit (Subbian et al., 2011) and rat (Singhal et al., 2011), which all display a course of disease that differs from human forms of TB. Mtb infection in nonhuman primates (Lin et al., 2010) closely resembles human TB, but this model is associated with high costs and, like any animal model, with ethical issues. Single-cell cultures of human macrophages or peripheral blood cells do not reflect the complex environment of human lung tissue. Thus, other well-defined experimental systems based on human cells are required to perform functional and mechanistic studies on TB under physiological conditions.

Here, we describe the formation of early TB granulomas in a recently established human lung tissue model (Nguyen Hoang et al., 2012). This culture system consists of tissue-specific epithelial cells and fibroblasts that form structures recapitulating normal human lung tissue, and is able to secrete mucus at the air-liquid interface (Nguyen Hoang et al., 2012). We show that this system allows implantation of human primary macrophages infected with mycobacteria and permits chemotactic migration of monocytes. To further validate our approach, we performed experiments with Mtb mutants lacking the RD1 region or ESAT-6. In consistence with previous *in vivo* (Davis and Ramakrishnan, 2009; Lewis et al., 2003; Pym et al., 2002; Volkman et al., 2004) studies, a functional ESX-1 was required for early granuloma formation. In addition, as recently described, ESAT-6 deficiency was associated with reduced ability of the bacteria to trigger granuloma formation (Volkman et al., 2010). The validated model provides a unique, physiologically relevant environment, which is useful both for dissection of host-Mtb interactions and for the development of novel therapeutic strategies.

**RESOURCE IMPACT**

**Background**

Tuberculosis (TB), caused by the bacterium *Mycobacterium tuberculosis*, is one of the major causes of death due to infectious disease. *M. tuberculosis* evades and replicates within host cells, giving rise to granulomatous tubercular lesions primarily found in the lungs. It is important to understand the mechanisms behind mycobacterial virulence, disease development and host cellular response to devise effective diagnostic, therapeutic and preventive strategies. One major challenge in TB research is the lack of functional resemblance to human TB of the widely used animal models. Non-human primates exhibit the disease that most closely resembles that in humans; however, these are associated with high operational and maintenance costs. Therefore, human tissue models that recapitulate aspects of human TB are of immense interest both for basic research and drug development.

**Results**

In this study, an experimental human lung tissue model for studies of TB is presented. The uninfected lung tissue model was recently established (by two of the co-authors) using human primary macrophages and monocytes together with cell lines of lung epithelial pneumocytes and fibroblasts, which were cultured on a matrix of collagen-embedded fibroblasts. The authors now show that the system, which retains cellular characteristics of pulmonary tissue, can be implanted with human macrophages infected with mycobacteria. They report that macrophages cluster at the site of *M. tuberculosis* infection, reminiscent of early TB granuloma. Furthermore, they demonstrate that early granuloma formation is dependent on the virulence factor ESAT-6, which is secreted by the type VII secretion system, ESX-1. This finding is consistent with previous *in vivo* studies of TB infection.

**Implications and future directions**

This work describes a biomimetic *in vitro* tissue model to facilitate studies of human TB. The model, which accurately recapitulates the key clinical and cellular features of human TB, has the potential to be employed in mechanistic studies of TB pathophysiology and for screening of candidate drugs. Furthermore, the model allows the introduction and manipulation of one or more chosen cell types, and analysis by a range of techniques opens up new avenues of research. Importantly, in addition to applications for TB research and TB drug discovery, this methodology can be adapted for studies of other respiratory infections, and should aid in understanding host-microbe interplay in general.

## RESULTS

### Mtb infection of the experimental lung tissue model

**Fig. 1.**
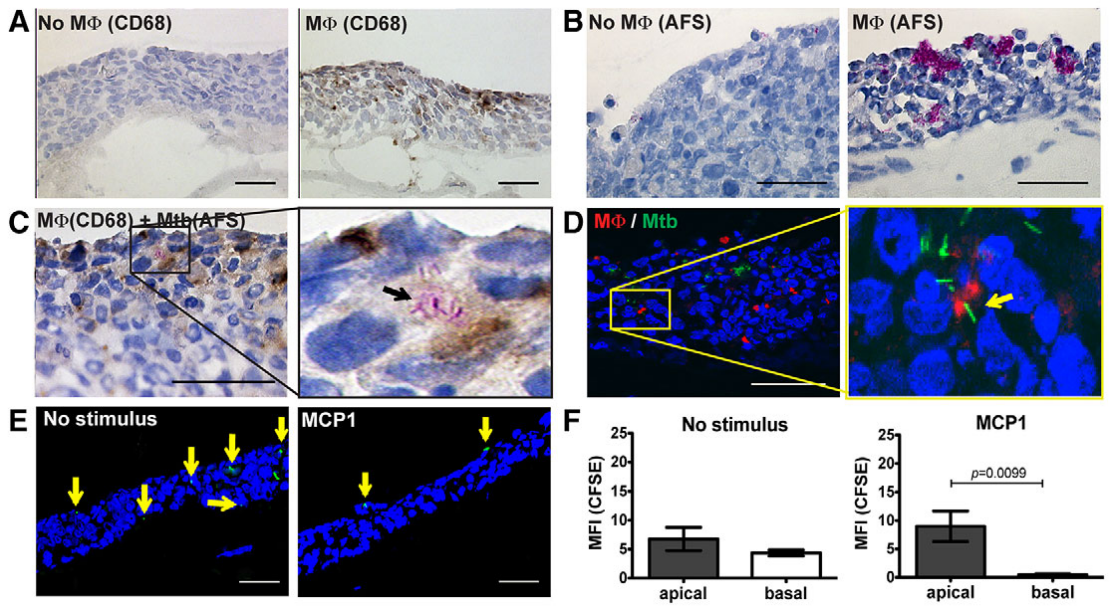
**Optimization of parameters for Mtb (H37Rv) infection of the lung tissue model.** (A) Immunohistochemistry of CD68-positive cells (brown) in tissue models without or with macrophages (MΦ). Cell nuclei are labeled with hematoxylin (HA, blue). (B) Apical Mtb infection of the tissue model without or with macrophages. Tissue sections were fixed and stained with HA (blue) and acid-fast staining (AFS, purple). (C) Mycobacterial infection of the tissue models using Mtb-infected macrophages as vehicles. Bright-field images of fixed sections stained with AFS (purple), immunohistochemistry (anti-human CD68, brown) and HA (blue). (D) Confocal images of Mtb-infected tissues. MΦ: anti-CD68 (red); Mtb: GFP (green); nuclei: DAPI (blue). The arrows in C and D indicate Mtb-infected macrophages. (E) CFSE-labeled monocytes (green) implanted in the model migrated towards the apical portion of the tissue in response to the monocyte-specific chemokine MCP1. Arrows in the image indicate green-labeled monocytes. (F) Quantitative analysis of the mean fluorescence intensity (MFI) in the apical versus basal layer of the tissues (means ± s.e.m., *n*=3). Statistical analysis was performed with a two-tailed unpaired *t*-test (F). Scale bars: 50 μm.

Using our established lung tissue model (Nguyen Hoang et al., 2012), we first optimized the introduction of macrophages derived from human donor blood into the experimental tissue. We found that addition of 50,000 macrophages/tissue model along with epithelial cells was the optimal approach to obtain an even distribution of macrophages as defined by CD68 expression (Fig. 1A). Tissues without macrophages were negative for CD68 staining (Fig. 1A). Although our success to infect the tissues with Mtb via the apical mucus layer was clearly macrophage-dependent, it resulted in excessive extracellular Mtb growth and aggregation (Fig. 1B). Therefore, as an alternative approach, we infected macrophages with Mtb before introducing them into the model at day 0 (D0), along with epithelial cells. Histological analysis revealed macrophage-associated acid-fast bacteria in the tissues at day 7 (D7) (Fig. 1C). Association of Mtb with macrophages was further assessed by introducing macrophages infected with green fluorescent protein (GFP)-expressing Mtb and subsequent immunofluorescence-based detection of the macrophages in D7 tissues (Fig. 1D). Formation of granulomas requires migration of monocytes/macrophages towards the site of infection. Because we could not observe migratory behavior of the macrophages in the tissue (not shown), we analyzed whether freshly prepared monocytes would migrate in the tissue in response to a chemoattractant stimulus. Addition of monocyte chemotactic protein 1 (MCP-1) to the apical side of the experimental tissues resulted in significant translocation of the fluorescently labeled cells towards the chemotactic gradient, as evidenced by increased mean fluorescence intensity (MFI) of carboxyfluorescein succinimidyl ester (CFSE) fluorescence at the apical sides of the tissues after 18 hours of incubation, showing that monocytes are highly motile in the tissue (Fig. 1E,F). In order to determine whether the lung tissue model supports spontaneous differentiation of monocytes to macrophages, monocytes were implanted into the experimental tissue at D0. Tissue sections prepared at D7 were subjected to immunohistochemistry using anti-CD68 antibodies (supplementary material Fig. S1A) or immunofluorescence using PKH26-labeled monocytes along with fluorescent anti-CD68 (supplementary material Fig. S1B). Quantification of the ratio of CD68-positive cells to total PKH26-labeled cells revealed that D7 models contained 89% CD68-positive cells.

### Monocytes/macrophages cluster in response to Mtb infection and form granuloma-like structures

**Fig. 2.**
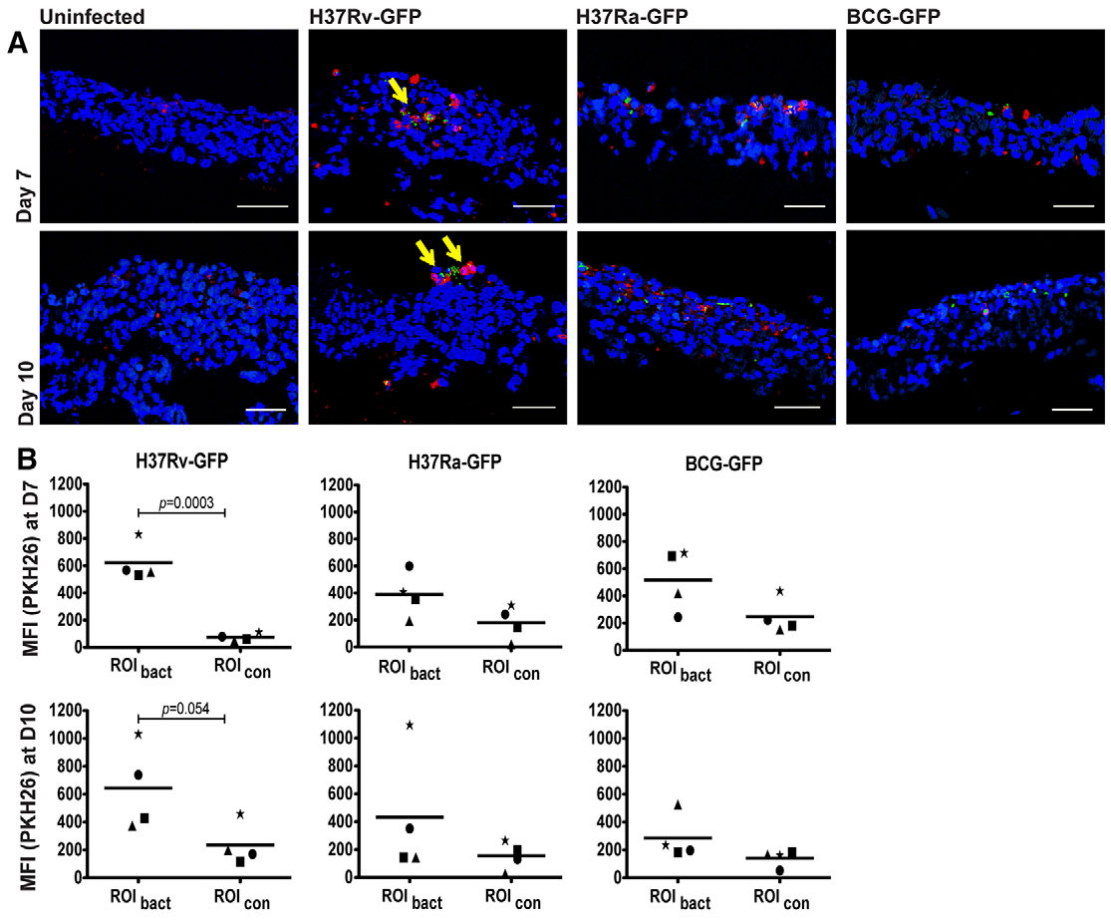
**Monocytes/macrophages in the tissue model cluster around virulent Mtb.** (A) Tissue models (nuclei stained with DAPI, blue) were implanted with PKH26-labeled monocytes (red) and macrophages infected with GFP-expressing (green) H37Rv, H37Ra or BCG, and sectioned. Fixed tissues were subjected to confocal microscopy at D7 or D10. Arrows indicate early granuloma structures. (B) Regions of interest (ROIs) with (ROI_bact_) and without (ROI_con_) bacteria were selected in the green channel and the MFI in the red channel was determined to quantify the recruitment of PKH26-labeled cells to the site of infection (*n*=4). Statistical analysis was carried out using a two-tailed unpaired *t*-test. Scale bars: 50 μm.

Initiation of granuloma formation, defined as the clustering of inflammatory macrophages, is an early event during mycobacterial infection (Davis and Ramakrishnan, 2009). To explore early granuloma formation in the lung tissue model, Mtb-infected macrophages and uninfected PKH26-labeled monocytes were introduced simultaneously at a ratio of 1:5 and the tissue was allowed to differentiate during air exposure, resulting in stratification of the epithelium (Nguyen Hoang et al., 2012). Confocal microscopy of sections of tissues fixed at D7 and D10 revealed that, in the presence of virulent Mtb (H37Rv) infection, monocytes were forming clusters at the site of infection (Fig. 2A). To quantify aggregation of the PKH26-labeled monocytes (macrophages), we measured the MFI from the red channel in manually selected regions of interest (ROI) with bacteria (MFI_bact_) and the MFI in an adjacent ROI without bacteria (MFI_con_) in the same microscopy field (internal controls). To ensure unbiased analysis, the ROIs were selected in the green channel (Mtb) and then the MFI (monocyte) was measured in the red channel in the same ROI. Analysis showed that the MFI in areas at sites with H37Rv infection differed significantly at D7 from the MFI of uninfected areas (Fig. 2B). This difference was not observed with the non-virulent strains H37Ra or BCG, or for any of the infecting strains at D10. The experiment was performed with two different MOIs (MOI 10 and 20) and MOI 10, which yielded the strongest clustering (data not shown), was therefore selected for further experiments.

### ESAT-6 secretion is required for early granuloma formation in the experimental model

**Fig. 3.**
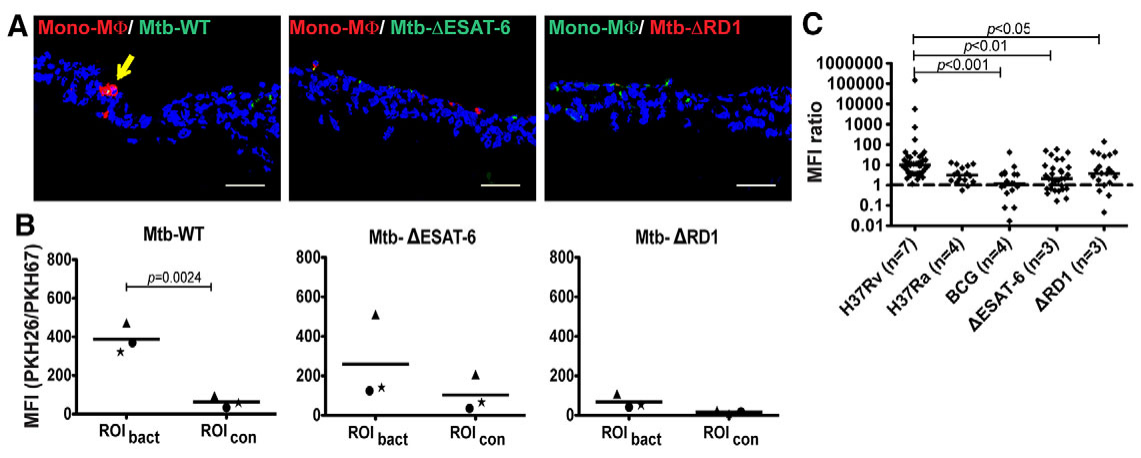
**RD1 and ESAT-6 are required for granuloma formation.** (A) Analysis of clustering of PKH26-labeled cells in D7 tissue models infected with wild type (WT) Mtb H37Rv, ΔESAT-6 (GFP) and ΔRD1 (mCherry). Arrow indicates a nascent granuloma. (B) Quantification of MFI of PKH26 (WT and ΔESAT-6) and PKH67 (ΔRD1) at sites with bacteria (ROI_bact_) and without bacteria (ROI_con_) (*n*=3). Statistical analysis was performed by using a two-tailed unpaired *t*-test. (C) Combined analysis of MFI_bact_:MFI_con_ ratios of the experiments with H37Rv, H37Ra, BCG, ΔESAT-6 and ΔRD1 (*n*=3–7). The solid lines represent the median ratios. Statistical analysis was performed with the Kruskal-Wallis test followed by Dunn’s multiple comparison test. Scale bars: 50 μm.

Because no gold standard method for Mtb-induced granuloma formation exists, we explored the fact that components of the RD1 region are required for granuloma formation (Davis and Ramakrishnan, 2009; Stoop et al., 2011; Volkman et al., 2004). Thus, applying a more qualitative approach to validate our model as representative of early granuloma formation, we introduced Mtb strains deficient in RD1 (∆RD1) or ESAT-6 (∆ESAT-6) into the experimental tissue. Clustering of monocytes/macrophages was determined by assessment of MFI of the stained cells at the site of infection. In line with the earlier studies, our analysis showed that the ∆RD1 strain was unable to induce clustering of monocytes/macrophages (Fig. 3A). We also confirmed that the ∆ESAT-6 strain was unable to cause clustering of monocytes at the sites of infection, further underlining the involvement of this virulence mechanism in the formation of early granulomas (Fig. 3A).

We took advantage of the fact that the same area was used for the measurement of each individual MFI_bact_ and MFI_con_, which allows compensation of varying basal staining intensities from the different sets of experiments. Thus, by calculating the MFI_bact_:MFI_con_ ratios from each individual measurement (described in more detail in the Materials and Methods section and supplementary material Fig. S2), we could perform a statistical analysis of all experiments together. At D7, the MFI ratio of tissues infected with H37Rv was significantly higher compared with the ratios from tissues infected with BCG, the ∆RD1 and the ∆ESAT-6 strains (Fig. 3B). Using this approach, the MFI ratio of H37Rv-infected tissues did not differ significantly from H37Ra (Fig. 3B).

**Fig. 4.**
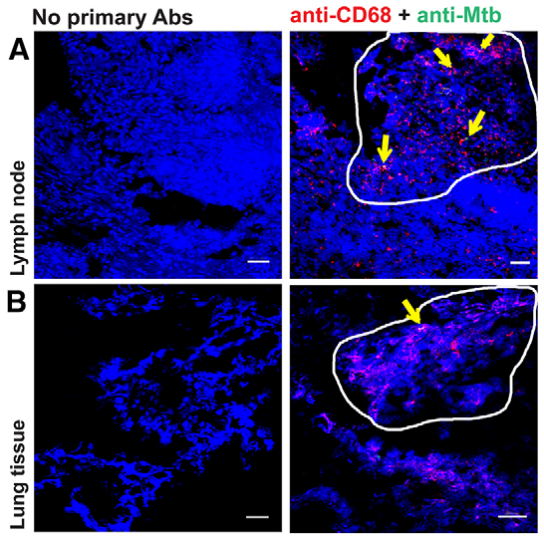
**TB-infected human lymph node and lung tissue sections display macrophage aggregates at sites with Mtb.** Sections of lymph node or lung biopsies obtained from TB patients were stained with anti-human CD68 (red), anti-Mtb (green) and DAPI (blue), and analyzed by confocal microscopy. Representative images from one of two analyzed lymph node (A) and lung tissue (B) biopsies from TB patients are shown. The area of the image enclosed by a solid line refers to a granulomatous region with macrophage aggregates and the arrows indicate macrophages harboring Mtb. Sections stained without primary antibodies (Abs) were used as controls. Scale bars: 50 μm.

The granuloma in our model represents early clusters of macrophages in Mtb-infected tissue and, to compare the appearance of our experimental granuloma with human TB granuloma, we visualized macrophages and Mtb in human lymph node and lung tissue biopsies obtained from Mtb-infected patients (Fig. 4).

### Infection with virulent Mtb causes necrosis in the experimental lung tissue model

**Fig. 5.**
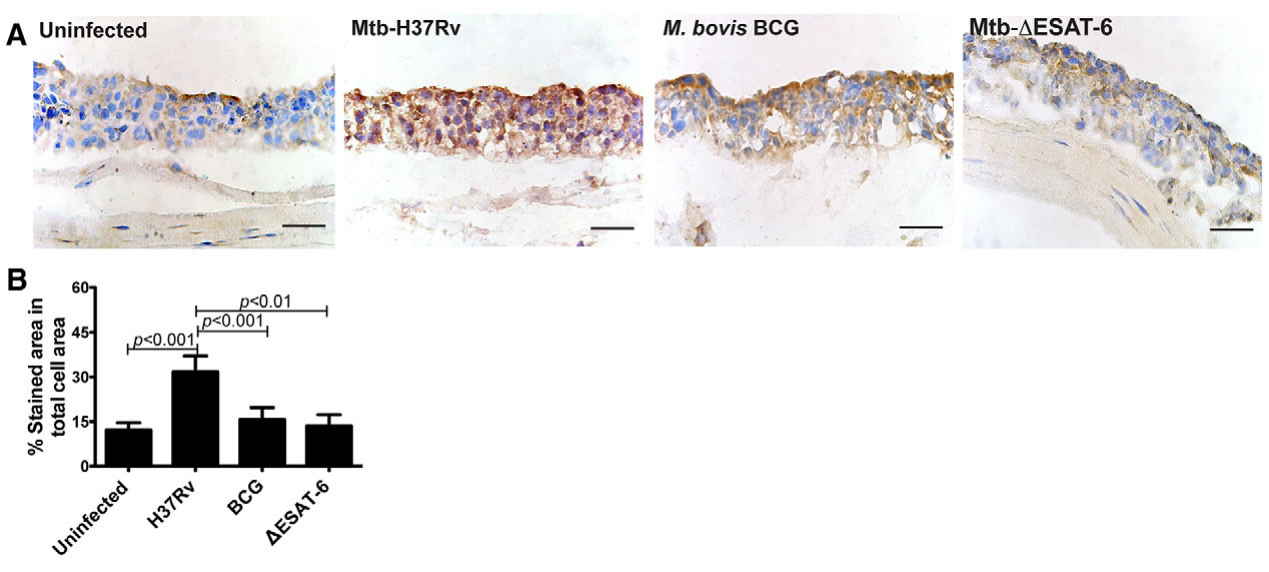
**Necrosis occurs in tissue models infected with virulent Mtb.** (A) The tissue models were infected with H37Rv, BCG or H37Rv–ΔESAT-6 and sectioned, fixed and stained for HMGB-1 (brown) and HA (blue) at D7. (B) *In situ* quantification of the HMGB-1 strain in A. Data are given as percentage of stained area out of total cell area and given as medians with interquartile range (*n*=3). Statistical analysis was performed with one-way ANOVA followed by Tukey’s multiple comparison test. Scale bars: 50 μm.

Uncontrolled Mtb infection is known to cause necrosis in human tissues (Brighenti and Andersson, 2012; Russell et al., 2009). To determine whether Mtb infection causes necrosis in the tissue model, we assessed *in situ* expression of the high mobility group box 1 (HMGB-1) protein, which is released from cells that undergo necrosis (Scaffidi et al., 2002). To this end, HMGB-1 was stained in sections of uninfected or H37Rv-, BCG- or ∆ESAT-6-infected tissues. Microscopy analysis and computerized quantification of HMGB-1 release in the tissue models revealed a significantly higher HMGB-1 staining in tissues infected with H37Rv compared with uninfected tissue or tissues infected with the non-virulent or mutant Mtb strains (Fig. 5).

## DISCUSSION

Of the earlier reported peripheral blood mononucleated cell (PBMC)-based *in vitro* models for TB granuloma formation (Kapoor et al., 2013; Puissegur et al., 2004), none included lung-tissue-specific cells and/or any three-dimensional scaffolding microenvironment representative of human lung tissue. To better reflect the *in vivo* conditions of human TB, we established Mtb infection in an *in vitro* lung tissue model based on human cells. Our lung tissue model displays a stratified epithelium supported by a fibroblast layer and is capable of mucus secretion and active formation of extracellular matrix proteins (supplementary material Fig. S3) (Nguyen Hoang et al., 2012). Using Mtb-infected macrophages as a physiologically relevant vehicle, we were able to establish a productive Mtb infection in this lung tissue model. Our optimization of the number and differentiation state of monocytes/macrophages, route and load of Mtb infection, and time for analysis provided the necessary details for the conditions under which TB granuloma formation can be studied.

The described *in vitro* model shares several characteristic features with the TB granuloma found in lung and lymph node tissue biopsies from TB patients, including aggregation of macrophages, the presence of both intra- and extracellular bacteria (Rahman et al., 2009; Ulrichs et al., 2004), and the induction of necrosis (Brighenti and Andersson, 2012; Russell et al., 2009). We observed that, in contrast to the virulent Mtb, strains defective in the ability to secrete ESAT-6 did not induce the clustering of monocytes/macrophages representative of early granuloma formation. This is in line with the observations made in zebrafish embryos, whose transparency allows live imaging of granuloma formation (Davis and Ramakrishnan, 2009). In that study, like in ours, the role of innate immunity during early granuloma formation was explicitly addressed, because these experiments were performed at a time point when the fish larvae had not yet developed adaptive immunity. As compared with the developed granuloma induced by virulent Mtb, BCG infection only modestly contributes to granuloma formation in the guinea pig model (Lagranderie et al., 1993), further supporting the involvement of RD1 components in this process. The correlation of this virulence determinant with the ability of mycobacteria to trigger granuloma formation suggests that, at least at some stage(s), the granuloma structure is exploited by the pathogen rather than being a host protective mechanism. Another aspect of the granuloma found in human TB is the presence of necrosis in advanced granuloma (Saunders and Britton, 2007). We have previously shown that Mtb-infected primary human macrophages undergo ESAT-6-dependent necrosis but not pyroptosis or pyronecrosis (Welin et al., 2011a). In a murine model of *M. marinum* infection, the RD1 region was shown to be responsible for tissue damage through necrosis (Carlsson et al., 2012). In line with these observations, we found necrosis only with wild-type virulent Mtb and not in the strains incapable of secreting ESAT-6.

Although the described experimental lung tissue only includes macrophages and monocytes besides lung-specific epithelial cells and fibroblasts, other immune cells can be introduced in the model. Here, the addition of certain lymphocyte subsets and neutrophils will be of great interest to continue to study the multifaceted response in TB infection. Importantly, the present model provides a relevant tissue environment not only for studies on TB, but for a variety of infectious and non-infectious diseases that affect the lungs. Potential uses of the lung model in basic and applied research include the study of innate immunity in TB or other lung infections, investigating mechanistic aspects of host defenses such as phagosomal maturation, autophagy and the production of cytokines, chemokines and antimicrobial effector molecules. In addition, the lung tissue model could be explored as a suitable tool for pharmacological or toxicological studies and also for evaluation of novel diagnostics and drug candidates. It is possible that the individual cell types, when assembled in tissue under the influence of stromal cells, behave differently compared with single-cell cultures. Tissue regulation of the immune response is a yet-unexplored field, which needs further attention to understand immunity in a complex, physiological environment. New insights into how virulence factors contribute to the establishment of infection, disease progression and dissemination, and host mechanisms required to restrict the invading pathogen, could be key to develop new therapeutic strategies to fight infectious disease.

## MATERIALS AND METHODS

### Ethics

Human peripheral blood from healthy anonymous blood donors purchased at the blood bank of Karolinska University Hospital, Karolinska Institute, Sweden was used as the source of immune cells for this study. Ethical permission for the use of these cells and for TB patient tissue biopsies was obtained from the regional ethics committee at Karolinska Institute, Stockholm, Sweden.

### Bacterial strains and growth conditions

The mycobacterial strains *M. tuberculosis* H37Rv wild type and ΔESAT-6, *M. tuberculosis* H37Ra and *M. bovis* BCG were transformed with the pFPV2 plasmid constitutively expressing GFP. The H37Rv-ΔRD1 mutant strain carried the plasmid pCherry3 expressing the red fluorescent protein mCherry. The bacteria were grown in Middlebrook 7H9 medium (Difco, Detroit, MI) supplemented with albumin, dextrose and catalase enrichment (Difco, Detroit, MI), 0.05% Tween-80 and (all except *M. bovis* BCG) with 0.5% glycerol at 37°C with 5% CO_2_ for 7–10 days. All the GFP-expressing strains were cultivated in the presence of kanamycin (20 μg/ml, Sigma-Aldrich, St Louis, MO), whereas hygromycin B (100 μg/ml, Sigma-Aldrich, St Louis, MO) was used rather than kanamycin for ΔRD1-mCherry. Experiments with virulent strains and mutants of Mtb were performed in a BSL-3 facility at the Swedish Institute for Infectious Disease Control (SMI), Stockholm, whereas avirulent strains were handled in a BSL-2 safety cabinet at the Centre for Infectious Medicine, Karolinska Institute, Sweden.

### Cell lines

MRC-5, a human lung fibroblast cell line (ATCC#CCL-171) derived from normal lung tissue of a 14-week-old male fetus, was maintained in complete Dulbecco’s modified Eagle’s medium (DMEM; Invitrogen), which includes supplementation of 1 mM sodium pyruvate, 2 mM L-glutamine, 100 U/ml penicillin, 100 μg/ml streptomycin, 10 mM HEPES, 0.1 mM non-essential amino acids and 10% heat-inactivated fetal bovine serum (FBS) (all from Invitrogen, Carlsbad, CA). The fibroblasts were used at passages 24–26 and grown until 70–80% confluency (7–9 days) before passaging.

The cell line 16HBE14o- (16HBE; a gift from Dr Dieter Gruenert, Mt Zion Cancer Center, University of California, San Francisco, CA) is an immortalized human bronchial epithelial cell line that was generated by transformation of human surface epithelial cells from a 1-year-old male with SV40 large T-antigen (Cozens et al., 1994), which retains the differentiated morphology and function of normal human airway epithelia. The 16HBE cells were cultured in fibronectin/collagen-coated flasks and maintained in complete Minimum Essential Medium Eagle (MEM, Sigma-Aldrich, St Louis, MO) supplemented with Earle’s salts, 0.292 g/l L-glutamine, 2.2 g/l sodium bicarbonate, 100 U/ml penicillin, 100 μg/ml streptomycin and 10% heat-inactivated FBS (Invitrogen) in 5% CO_2_ at 37°C.

### Isolation and culture of monocytes

Peripheral blood monocytes from donor blood were isolated using RosetteSep (Stemcell Technologies, Vancouver, BC, Canada) followed by lymphoprep density gradient centrifugation. Cells were washed twice with phosphate buffered salt solution (PBS), resuspended at a density of 2×10^6^ cells per ml in complete DMEM for direct use as monocytes or in complete DMEM supplemented with 50 ng/ml recombinant human monocyte colony stimulating factor (Stemcell Technologies, Vancouver, BC, Canada) and cultured 7 days at 37°C for differentiation into macrophages.

### Mtb infection of macrophages

The cultured bacteria were harvested, washed with PBS containing 0.05% Tween 80, resuspended in antibiotic-free complete DMEM, passed through a 27-gauge needle to disperse bacterial clumps and the optical density was measured. The macrophages were infected overnight with mycobacteria at MOI 10. The extracellular bacteria in the macrophage culture were removed by washing thrice with PBS. The macrophage cultures were washed with PBS to remove extracellular bacteria. The infected macrophages were detached by treatment with 2 mM EDTA (Sigma-Aldrich, St Louis, MO) for 10 minutes at 37°C and resuspended in antibiotic-free complete DMEM.

### Labeling of monocytes

To determine monocyte migration in the model, monocytes were labeled with 5 μM CFSE dye (Invitrogen, Carlsbad, CA) for 30 minutes. To assess the immune cell cluster formation in response to Mtb infection by different strains, monocytes labeled with PKH26 or PKH67 (Sigma-Aldrich, St Louis, MO) were introduced into the tissue model. Briefly, freshly prepared monocytes (1×10^7^ cells) were stained with 2 μM PKH26 or PKH67 for 5 minutes according to the manufacturer’s instructions and washed 3× with serum-supplemented, antibiotic-free complete DMEM before the transfer into the model.

### The lung tissue model

The *in vitro* lung tissue model is a development of Dongari-Bagtzoglou and Kashleva (Dongari-Bagtzoglou and Kashleva, 2006) and modification of Nguyen Hoang et al. (Nguyen Hoang et al., 2012). In this model, human lung epithelial type II pneumocytes and human primary macrophages/monocytes were seeded into a matrix of collagen-embedded fibroblasts. Briefly, an acellular collagen layer (bovine type I collagen that was treated with phosphoric acid, Organogenesis; 200–055) is first formed on six-well transwell filters with 3 μM membranes (BD Biosciences, San Jose, CA) and kept at 37°C. A collagen-embedded fibroblast layer was added to the acellular layer and incubated for 2 hours at 37°C before adding the complete medium in the outer well. Complete medium was added to the inner well the next day and incubated for 4–6 days to allow gel contraction. The contracted tissue displays a raised conical structure in the center of the well and is detached completely from the wall of the insert. Uninfected and infected macrophages and freshly prepared monocytes labeled with red (PKH26) or green (PKH67) dye (Sigma-Aldrich, St Louis, MO) mixed at a 1:5 ratio were seeded onto the contracted tissue along with epithelial cells at a density of 10^5^ cells per model. After an additional 2–3 days of culturing, the models were exposed to air by removing the medium from the inner chambers and reducing the medium in the outer chambers to 1.5 ml. After the experiment, the filters with the tissue models were cut out of the chambers and embedded in Tissue-Tek OCT-containing cassettes (both from Sakura Finetek, AJ Alphen aan den Rijn, The Netherlands), frozen in liquid nitrogen and stored at −80°C. The frozen tissues were sectioned using a Microm HM560 Cryo-Star cryostat (Thermo Scientific, Erembodegem, Belgium), and the tissue sections were collected on Superfrost slides (Thermo Scientific, Erembodegem, Belgium) and fixed with 4% formaldehyde (BD Biosciences, CA). The slides with the tissue sections were either mounted directly in Prolong Gold antifade with DAPI (Invitrogen, Carlsbad, CA) or processed for staining by immunohistochemistry or immunofluorescence.

### Localization of macrophages and Mtb in the tissue model

Cryosections (8 μm) of the tissue models were stained as described (Nguyen Hoang et al., 2012) with anti-human CD68 (Dako, Glostrup, Denmark) overnight at room temperature followed by a biotinylated anti-mouse antibody (Ab; Dako, Glostrup, Denmark). Endogenous avidin, biotin and catalase activity in the sections was neutralized before incubation with the primary Abs. Tissue sections were also blocked with normal serum before incubation with secondary Abs, to prevent non-specific binding. The secondary Ab was detected with the peroxidase-based Vectastain Elite ABC kit (Vector Laboratories, Burlingame, CA), and the reaction developed with the diaminobenzidine tetrahydrochloride (DAB) peroxidase substrate kit (Vector Laboratories, Burlingame, CA). Cell nuclei were counterstained with Mayer’s hematoxylin (HA; Histolab Products, Gothenburg, Sweden). Tissue sections from models without macrophages were used as negative controls. Acid-fast staining (AFS) was subsequently performed using the Kinyoun’s staining protocol. The slides were washed, dried and mounted for analysis. For immunofluorescence, tissue sections from models infected with GFP-expressing Mtb were stained with anti-human CD68 (Dako, Glostrup, Denmark) followed by goat anti-mouse Alexa Fluor 594 (Invitrogen, Carlsbad, CA) and mounted directly in Prolong Gold antifade with DAPI (Invitrogen, Carlsbad, CA).

### Migration assay

Tissue models implanted with CFSE-labeled monocytes (green) were air-exposed for 3 days to allow stratification of apical layers and mucus secretion. The apical surfaces of these models were treated (or left untreated) with 100 ng/ml of MCP1 (PeproTech, London, UK) dissolved in complete DMEM (Fig. 1E). The models were harvested, frozen, sectioned and analyzed by confocal microscopy (Nikon A1 confocal system). For quantification of monocyte migration, tissue regions were first selected in the blue channel and then were divided into apical and basal regions, and finally the MFIs for apical and basal regions were determined in the green channel (NIS Elements AR image analysis software, Nikon Instruments, Melville, NY) (Fig. 1F).

### Monocyte differentiation in the tissue model

Tissue models were implanted with 200,000 monocytes, cultured and harvested at D7. For immunohistochemistry, tissue sections were stained with anti-human CD68 (Dako, Glostrup, Denmark) overnight followed by blocking with serum and secondary Abs, which were detected and developed with the Vectastain Elite ABC and DAB kits, respectively (Vector Laboratories, Burlingame, CA). Cell nuclei were counterstained with hematoxylin (HA) (Histolab Products, Gothenburg, Sweden). Tissue sections stained with secondary Abs alone were used as negative controls. For immunofluorescence, models implanted with PKH26-labeled monocytes (red) were stained for anti-human CD68 (Dako, Glostrup, Denmark) followed by goat anti-mouse Alexa Fluor 488 (green). The ratio of CD68-positive fluorescence over PKH26-labeled cells was determined to assess the percentage of monocytes that had differentiated into macrophages (Zeiss LSM700 Confocal system and Zen image analysis software, Zeiss, Gottingen, Germany).

### Confocal microscopy and quantification of early granuloma formation

Sections of the lung tissue models were analyzed using a Nikon A1 confocal microscope (Nikon Instruments, Melville, NY). Images from 10–15 fields/tissue section were included in the analysis. The MFI of PKH26 (monocytes or monocytes that spontaneously differentiated into macrophages) in the ROI with bacteria (MFI_bact_) was compared with that in the ROI without bacteria (MFI_con_) from the same image field (internal controls) using the NIS Elements AR image analysis software (Nikon). To ensure unbiased analysis, selections of the ROIs were made in the green channel (Mtb) and then the MFI within the ROI was measured in the red channel (PKH26) (supplementary material Fig. S2). For the ΔRD1 strain (mCherry, red fluorescence), the ROIs were selected in the red channel and the measurement of the monocyte/macrophage aggregation was done in the green channel (PKH67). All the ROIs were from cellular regions of the tissue as identified in the blue channel (DAPI, cell nuclei). Because the area used for the measurement of MFI_bact_ and MFI_con_ was identical for individual measurement pairs (MFI_bact_ and MFI_con_), ratios could be calculated to better compare the recruitment of monocytes/macrophages in different experiments.

### Immunofluorescence staining of human TB tissues

Cryosections (8 μm) of human lymph node and lung tissue biopsies from TB patients were collected and fixed in a BSL-3 facility. Tissue sections were stained with anti-human CD68 (Dako, Glostrup, Denmark) overnight followed by goat anti-mouse Alexa Fluor 594 (Invitrogen, Carlsbad, CA). Sections were blocked with normal rabbit serum before incubating with rabbit polyclonal *M. bovis* BCG (Dako, Glostrup, Denmark) for 2 hours followed by swine anti-rabbit Alexa Fluor 488 (Invitrogen, Carlsbad, CA). The slides with the tissue sections were mounted directly in Prolong Gold antifade with DAPI (Invitrogen, Carlsbad, CA). Tissue sections treated with no primary Abs were considered as controls.

### Immunohistochemistry and *in situ* quantitative image analysis of HMGB-1

Cryosections of tissue models were stained as described (Nguyen Hoang et al., 2012) with polyclonal rabbit HMGB-1 (Abcam, Cambridge, UK) overnight at room temperature followed by biotinylated anti-rabbit secondary Ab (Dako, Glostrup, Denmark). Endogenous avidin, biotin and catalase activity in the sections was neutralized before incubation with the primary Abs. Tissue sections were also blocked with normal serum before incubation with secondary Abs, to prevent non-specific binding. The secondary Abs were detected, reaction developed and cell nuclei counterstained as described above. Tissue sections stained with no primary Abs were used as negative controls.

The tissue sections were analyzed using a Leica DMR-X microscope (Leica Microsystems GmbH, Wetzlar, Germany) coupled to a computerized image analysis system (Leica Qwin 5501W, Leica Microsystems GmbH, Wetzlar, Germany) as described (Rahman et al., 2012). Protein expression was quantified according to the following procedure: digital exclusion of tissue artifacts; threshold for the intensity of positive staining; threshold for the total cell area by including both positive (DAB) and negative (HA) staining; and application of a determined setting for 10–15 fields/tissue section. Data are obtained as the mean percentage of positively stained area within the total cell area of a specific marker.

## Supplementary Material

Supplementary Material
