# Supplementary material for: Modeling Mycobacterium tuberculosis early granuloma formation in experimental human lung tissue
Source: Dis Model Mech. 2013 Nov 7;7(2):281–8. doi: 10.1242/dmm.013854 (PMC3917249; doi:10.1242/dmm.013854)
Supplement: Supplementary Material [file supp_7_2_281__index.html]

Modeling Mycobacterium tuberculosis early granuloma formation in experimental human lung tissue — Supplementary Material 

# Modeling *Mycobacterium tuberculosis* early granuloma formation in experimental human lung tissue

## DMM013854 Supplementary Material

**Files in this Data Supplement:**

- **Supplementary Material PDF**
