# Supplementary material for: Modeling Mycobacterium tuberculosis early granuloma formation in experimental human lung tissue
Source: Dis Model Mech. 2013 Nov 7;7(2):281–8. doi: 10.1242/dmm.013854 (PMC3917249; doi:10.1242/dmm.013854)

Fig. S1. The tissue models support monocyte differentiation. (A) Bright field images of sections of tissue models implanted with freshly prepared peripheral blood monocytes stained with HA (blue) and anti-CD68 (brown) (D7). (B) Confocal images of sections of uninfected tissue models implanted with PKH26-labeled monocytes (red) and stained post-fixation with anti-CD68 (green). Sections stained without primary antibodies (Abs) were used as controls in (A) and (B). Scale bars are 50μm.

Supplementary Fig. S1

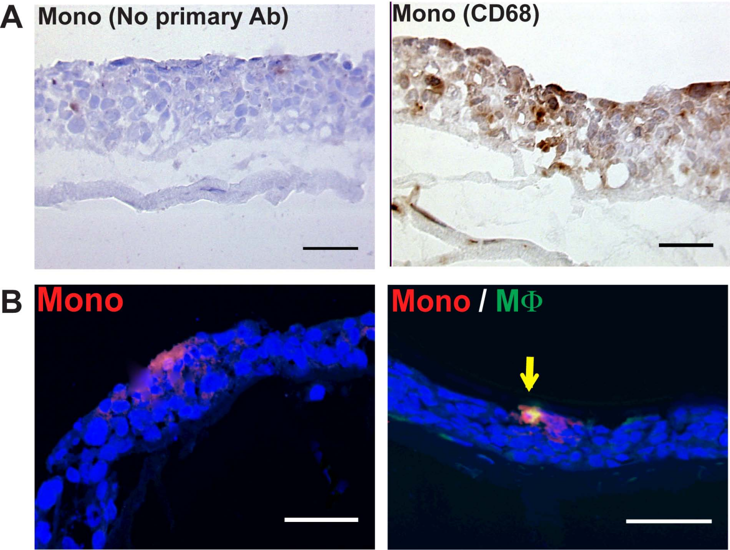

Fig. S2. Quantification of monocyte (Mono)/macrophages (MΦ) clustering at the site of Mtb infection. DAPI (blue)-stained sections of infected tissue models implanted with PKH26-labeled monocytes (red) and GFP-expressing mycobacteria (green) were analyzed by confocal microscopy. Regions of interest with bacteria (ROI_{bact}) were selected in the green channel (B) and the Mean Fluoresence Intensity (MFI) was measured in this region in the red channel (MFI_{bact}, (C); PKH26-expressing monocytes/macrophages). The same ROI was moved to a place without infection (ROI_{con}) and the MFI in the red channel was measured (MFI_{con}, D). Results are presented either as the absolute values of the MFIs or as ratios of the individual MFI_{bact}/MFI_{con} pairs. Scale bars are 50 µm.

Supplementary Fig. S2

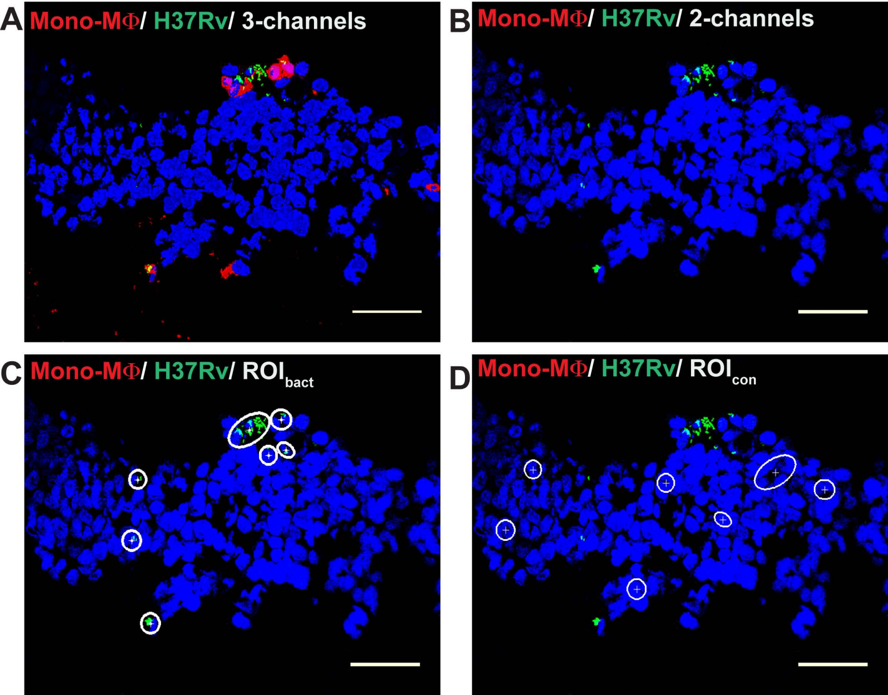

Fig. S3. The human in vitro lung tissue model for TB infection. The tissue model involves addition of immune cells and human bronchial epithelial cells onto the human lung fibroblasts embedded in a collagen matrix created on a transwell membrane. Mtb (green)-infected macrophages (yellow) are used as vehicles to introduce the infection into the tissue. Pre-labeled monocytes (red) are added to visualize recruitment of this cell type to the site of infection. The tissue models are air-lifted to ensure stratification of the epithelia and mucus secretion. The model developed by this method enables the investigation of early TB granuloma formation.

Supplementary Fig. S3

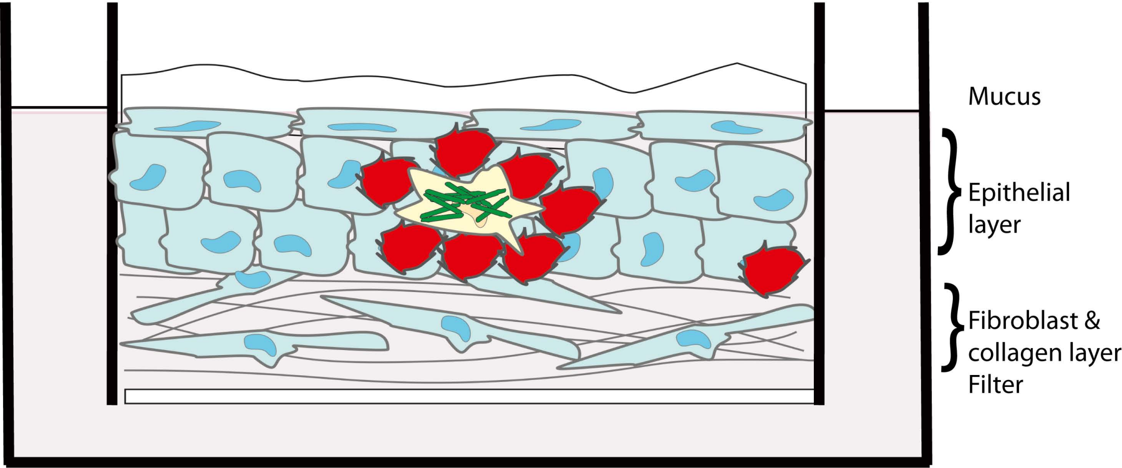

Supplement: Supplementary Material [file supp_7.2.281_DMM013854.pdf]
